# Effect of a telemedicine intervention for diabetes-related foot ulcers on health, well-being and quality of life: secondary outcomes from a cluster randomized controlled trial (DiaFOTo)

**DOI:** 10.1186/s12902-020-00637-x

**Published:** 2020-10-21

**Authors:** Marjolein M. Iversen, Jannicke Igland, Hilde Smith-Strøm, Truls Østbye, Grethe S. Tell, Svein Skeie, John G. Cooper, Mark Peyrot, Marit Graue

**Affiliations:** 1 Faculty of Health and Social Sciences, Department of Health and Caring Sciences, Western Norway University of Applied Sciences, N-5020 Bergen, Norway; 2 Department of Medicine, Section of Endocrinology, Stavanger University Hospital, Stavanger, Norway; 3 Department of Global Public Health and Primary Care, University of Bergen, Bergen, Norway; 4 Duke Global Health Institute, Duke University, Durham, NC USA; 5 Department of Research, Stavanger University Hospital, Stavanger, Norway; 6 Department of Sociology, Loyola University Maryland, Baltimore, MD USA

**Keywords:** Diabetic foot, Psychological aspects, Health care delivery, Clinical trials

## Abstract

**Background:**

Follow-up care provided via telemedicine (TM) is intended to be a more integrated care pathway to manage diabetes-related foot ulcers (DFU) than traditionally-delivered healthcare. However, knowledge of the effect of TM follow-up on PROMs including self-reported health, well-being and QOL in patients with DFUs is lacking and often neglected in RCT reports in general. Therefore, in this study of secondary outcomes from the DiaFOTo trial, the aim was to compare changes in self-reported health, well-being and QOL between patients with DFUs receiving telemedicine follow-up care in primary healthcare in collaboration with specialist healthcare, and patients receiving standard outpatient care.

**Methods:**

The current study reports secondary endpoints from a cluster randomized controlled trial whose primary endpoint was ulcer healing time. The trial included 182 adults with diabetes-related foot ulcers (94/88 in the telemedicine/standard care groups) in 42 municipalities/districts, recruited from three clinical sites in Western Norway. Mean (SD) diabetes duration for the study population was 20.8 (15.0). The intervention group received care in the community in collaboration with specialist healthcare using an asynchronous telemedicine intervention. The intervention included an interactive web-based ulcer record and a mobile phone enabling counseling and communication between the community nurses and specialist healthcare; the control group received standard outpatient care. In total 156 participants (78/78) reported on secondary endpoints: self-reported health, well-being and quality of life evaluated by generic and disease-specific patient-reported outcome measures (e.g. Euro-QOL, the Hospital Anxiety and Depression Scale (HADS), Problem Areas in Diabetes (PAID), Neuropathy and Foot Ulcer–Specific Quality of Life Instrument (NeuroQOL)). Linear mixed-effects regression was used to investigate possible differences in changes in the scores between the intervention and control group at the end of follow-up.

**Results:**

In intention to treat analyses, differences between treatment groups were small and non-significant for the health and well-being scale scores, as well as for diabetes-related distress and foot ulcer-specific quality of life.

**Conclusions:**

There were no significant differences in changes in scores for the patient reported outcomes between the intervention and control group, indicating that the intervention did not affect the participants’ health, well-being and quality of life.

**Trial registration:**

Clinicaltrials.gov, NCT01710774. Registered October 19th, 2012.

## Background

Follow-up care provided via telemedicine (TM) is intended to be a more integrated care pathway to manage diabetes-related foot ulcers (DFU) than traditionally-delivered healthcare. However, TM follow-up in diabetes care can alter patient-provider relationships, and might have unintended negative effects on health, well-being and quality of life (QOL) [1]. Hence, it is important to also evaluate self-reported health and well-being outcomes among those receiving telemedicine follow-up care. Measures of health-related quality of life (HRQOL) among adults living with diabetes-related foot ulcers have shown that the occurrence of a DFU, in general, is associated with reduced quality of life [2–4]. While several different patient-reported outcome measures (PROMs) have been used, none has been found to be more appropriate than others in assessing HRQOL associated with diabetes-related foot ulcers [3, 4]. Although generic measures such as the WHO-Five Well-Being Index (WHO-5) and the Euro-QOL five-dimension questionnaire (EQ-5D-5L) are useful, they do not capture disease-specific aspects of HRQOL [4]. Disease or condition specific PROMs, such as Problem Areas in Diabetes (PAID) and Neuropathy and Foot Ulcer–Specific Quality of Life (NeuroQOL) may be more useful to study foot ulcer patients’ distress and their response to treatment [5]. Moreover, identifying emotional distress in foot ulcer patients with diabetes is important as depression is associated with development of the first foot ulcer [6] and symptoms of depression are associated with an increased risk of a diabetes-related foot ulcer in a dose response manner [7]. Analyses of PROMs utilizing validated instruments allow healthcare providers to determine whether different treatments provide patients with the best possible outcomes and improve HRQOL [4].

Two TM randomized controlled trials among adults with a DFU have been performed, one in Denmark [8] and one in Norway [9]. Both studies evaluated the effect of TM on ulcer healing, amputation and death and compared TM with SOC. The Danish study found no significant differences regarding amputation and healing time [8]. In the Norwegian study, the Diabetic Foot and Telemedical Images Project (DiaFOTo) [9], we confirmed the hypothesis that TM in patients with DFUs in primary healthcare, in collaboration with specialist healthcare, was non-inferior to standard outpatient care (SOC) regarding healing time among those whose DFU healed (79.8% vs 76.1%). The direction of the effect estimates for healing time in months (0.43 (− 1.50, 0.65)), death (− 0.4% (− 6.5, 5.7%)), and consultations all favored TM, although there were no statistically significant effects. Telemedicine monitoring was superior regarding avoidance of amputations (6.4% vs 14.8%) in the Norwegian study. However, knowledge of the effect of TM follow-up on PROMs (including self-reported health, well-being and QOL) in patients with DFUs is lacking and often neglected in RCT reports in general. Therefore, in this study of secondary outcomes from the DiaFOTo trial, the aim was to compare changes in self-reported health, well-being and QOL between patients with DFUs receiving telemedicine follow-up care in primary healthcare in collaboration with specialist healthcare, and patients receiving SOC.

## Methods

### Trial design

The design of the study has been described previously [9, 10]. Briefly, this was a multicenter cluster-randomized controlled noninferiority trial. Patients from three clinical sites in western Norway were included (NCT01710774). The recruitment period lasted from September 2012 to June 2016. A total of 182 patients were included, 94 in the TM group and 88 in the control group in 42 municipalities/districts. Forty-two clusters were matched in 21 pairs which were then randomized to either TM or control. These were matched in pairs according to population size and rural/urban characteristics. The randomization sequences were generated by an independent statistician using SPSS version 21 statistical software (IBM Corp) [10]. The study was approved by the Western Norway Regional Committee for Medical and Health Research Ethics (2011/1609). All participants gave written consent. The study adheres to CONSORT guidelines (Additional file 1).

### Participants

Included in the original trial were patients with type 1 or type 2 diabetes who were 20 years or older, diagnosed with a new DFU [9], defined as a skin lesion below the ankle. Excluded were patients who had an ulcer on the same foot treated during the last 6 months in specialist healthcare or a diagnosis of mental disorder or cognitive impairment. Another exclusion criteria was having a life expectancy less than 1 year, and the patient also had to be able to understand and write Norwegian [10]. Patients in both groups were followed until ulcer healing, amputation, or death, up to a maximum of 12 months of follow-up. (The Consort flow chart for the primary study is available as Fig. S1 in the supplementary appendix.)

In the current study we included all participants with at least one valid patient-reported outcome measure at baseline in the TM group (*n* = 78 (83.0%)) and in the control group (*n =* 78 (88.6%)). PROMs were collected at baseline and at predefined endpoints (healed ulcer or amputation or at 12 months if the ulcer did not heal). For persons who died during follow-up or were lost to follow-up for other reasons, only the baseline measurements were used in analyses and these persons did thus not contribute to the estimation of the intervention effect.

### Intervention

#### Telemedicine (TM)

The randomization procedures and a more in-depth description of the intervention have been reported previously [9]. Briefly, community nurses used a mobile phone to take pictures of the ulcer which was then sent via a web-based platform to the hospital for review by a specialist health care professional, facilitating counseling and feed-back. This platform is a web-based ulcer record system accessible from mobile devices and computers via the Internet [9–11]. The platform facilitate to register clinical data regarding the ulcers, digital images of the ulcers, a written assessment of the ulcer, and to ask for and give guidance. Measurements can be compared over time to visualize the healing process of the ulcer. This allows all involved staff to contribute, even though they are on a distance. This system is customized for collaboration, discussions, and advice regarding the treatment between cooperating medical staff in different health care institutions. The main reason for using the web-based ulcer record system is the collaboration functionality that enables integrated care across different levels of the health care sector. Relevant ulcer data are accessible to the involved staff, regardless of employment in community care or specialist health care. During follow-up, the community nurses provided care under supervision of the specialist nurses at the outpatient clinics and communicated at least weekly with the specialist nurses at the outpatient clinic [10].

#### Standard outpatient care

Patients in the control group received standard care provided by the outpatient clinic, normally scheduled to take place every second week. The medical treatment given to the control and intervention group is based on the same evidence-based procedures [10].

### PROMs

We used both generic and disease-specific PROMs to assess the patients’ perceptions of the intervention on self-reported health and well-being, and QOL. Included were three generic instruments (Euro-QOL EQ-5D-5L/ EQ-5D-VAS, the Hospital Anxiety and Depression Scale (HADS), the WHO-Five Well-Being Index (WHO-5)), and two disease-specific measures; Problem Areas In Diabetes (PAID-20) and Neuropathy and Foot Ulcer–Specific Quality of Life (NeuroQOL).

We used the EQ-5D-5L questionnaire as a health utility score [12]. Part one comprises five single items: mobility, self-care, usual activities, pain/discomfort and anxiety/depression (*dimensions of health)* with ratings on five levels of perceived problems from no problems (1) to extreme problems (5). The level scores are presented as global health indices with a weighted total value for health status [13]. The second part is a Visual Analogue Scale (VAS) ranging from 0 (worst health state) to 100 (best health state), and used as an overall measure of perceived health status [12]. The WHO-5 was used to describe *well-being* during the previous 2 weeks [14]. Five items with ratings from 0 to 5. Item scores are summed (0–25) and transformed to a 0–100 scale with higher scores indicating better well-being. The HADS assessed *anxiety and depressive symptoms* during the past week [15]. This instrument comprises seven items on anxiety (HADS-A) and seven items on depression (HADS-D). Each item is scored from 0 to 3, yielding a maximum score of 21. Higher scores indicate a higher symptom load.

*Diabetes distress* was measured with PAID-20 [16, 17]. The instrument covers frequently reported emotional states. Scale scores are transformed to a 0–100 scale, with 100 indicating greater distress. The cut-off score is suggested to be equal or more than 40. The NeuroQOL assessed patients’ perception of *the impact of foot ulcers on their QOL* [18]. The instrument consists of six domains: 1) painfully symptoms and paresthesia; 2) symptoms of reduced/lost feeling in the feet; 3) diffuse sensory motor symptoms; 4) restrictions in daily activities; 5) interpersonal problems and 6) emotional burden. Higher scores indicate a greater negative impact of foot ulcers on QOL.

### Other measures

*Healing of the ulcer* was defined as healing (intact skin) of the whole foot without minor or major amputations. *Amputation* was defined as minor or major amputation before ulcer healing. Amputation performed below the ankle was defined as minor amputation, whereas amputation above the ankle was defined as major amputation. *Death* was defined as death before ulcer healing.

In addition, clinical data, comorbidities and foot ulcer specific data were collected from the electronic medical journals at the clinical sites. Those with grade 2 or grade 3 combined with stage C and D were defined as high severity and all other grades and stages were combined to a group with versus low and medium severity [19]. Other measures as demographic data and lifestyle characteristics were self-reported by the patient [10] (Table 1).

### Statistical analyses

Data were analyzed according to the initial group allocation (intention to treat). Continuous variables are presented as mean and standard deviations (SD), and categorical variables are presented as proportions. We used linear mixed effects regression analyses with random intercept to account for clustering within treatment groups. Intra-cluster correlation for the primary outcome healing time in the main study was 0.0014. All models were specified with the sum score of the PROMs (EQ-5D-5L, HADS, WHO-5, PAID-20 and the NeuroQOL subscales respectively) as dependent variables with fixed effects for time, group allocation and interaction between time and allocation group. The coefficient for the interaction term between time and allocation group was reported as the intervention effect and can be interpreted as the difference in change in PROM score between the two groups after adjustment for baseline differences in PROM score. The intervention effects are reported as regression coefficients with 95% CIs. For analyses of the dichotomous outcome PAID> 40 we used generalized estimation equations (GEE) instead of linear mixed models. Sample size calculation was reported previously [9].

### Additional analyses

In additional analyses, we tested whether distance to the outpatient clinic moderated the intervention effect. We conducted a subgroup analysis with a linear mixed model, including only patients who lived 25 km from the outpatient clinic. All analyses were also repeated excluding 13 patients originally assigned to the TM group who did not receive TM (per protocol analyses) [9, 10]. A greater percentage of participants in the intervention group with ulcers on the toes than in the control group suggested possible differential selection. In sensitivity analyses we therefore repeated the linear mixed-effects regression analyses and GEE adjusted for localization of ulcer. We also did analyses with stratification on severity of ulcer measured in terms of grade and stage from the UT classification system [10, 20]. Statistical significance was defined as *P* < 0.05. Statistical analyses and graphs were performed and constructed in Stata (version 14).

## Results

### Trial participants

In total, 345 patients were assessed for eligibility between September 2012 and June 2016; of these 163 did not meet the inclusion criteria. In total 182 patients were included (94 in the TM group) and 88 in the SOC group) (Table 1). Most participants were male (73.7%), the mean HbA1c was 7.9% (SD 1.7), and the majority used insulin (65.2%). A history of cardiovascular disease and neuropathy was present in 27.3 and 70.0% of patients, respectively. Furthermore, most ulcers were classified as grade 1 and stage A or B at baseline [9]. Overall, characteristics were well balanced between the two groups at baseline, except type of diabetes and localization of ulcer. Completion rates for PROMs for patients with at least one valid PROM at baseline were 87% (Table S1 in the supplementary appendix) (*n* = 156/182). Mean (SD) time between baseline measurement and follow-up measurement of PROMs was 4.4 months (4.1) in the TM group and 4.2 months (3.6) in the SOC group (Not shown in table). The mean (± SD) scores for PROMs at baseline and post-intervention and regression coefficients for the intervention effect are reported in Table 2. In addition, unweighted scores for the NeuroQOL at baseline and follow-up are reported in Table S2.

**Table 1:** Characteristics of the participants: the DiaFOTo study, Western Norway^a^

Characteristics	Total	Telemedicine	Standard outpatient care
	(*n* = 156)	*(n* = 78)	*(n* = 78)
*Demographic variables*			
Male sex	115 (73.7)	57 (73.1)	58 (74.4)
Age (years)	65.5 ± 16.3	66.3 ± 16.3	64.7 ± 16.4
Married or Cohabitant	91 (60.7)	42 (56.8)	49 (64.5)
Education level^b^			
Primary	36 (25.5)	22 (32.4)	14 (19.2)
Secondary	73 (51.8)	34 (50.0)	39 (53.4)
Tertiary	32 (22.7)	12 (17.7)	20 (27.4)
Employed	34 (21.9)	18 (23.4)	16 (20.5)
Travel distance > 25 km to hospital	40 (27.4)	21 (28.4)	19 (26.4)
Lifestyle characteristics			
Smoking (yes)	27 (18.1)	14 (18.9)	13 (17.3)
*Subgroups of diabetes*			
Type 1 diabetes	35 (22.4)	11 (14.1)	24 (30.8)
Type 2 diabetes	121 (77.6)	67 (85.9)	54 (69.2)
*Diabetes-related variables*			
Diabetes duration, years	20.8 ± 15.0	19.8 ± 14.1	21.8 ± 15.8
Insulin treatment	101 (65.2)	47 (61.0)	54 (69.2)
HbA_1c_, (mmol/mol)^c^	63 ± 18.6	63 ± 17.5	64 ± 18.6
HbA_1c_, (%)^b^	7.9 ± 1.7	7.9 ± 1.6	8.0 ± 1.7
*Ulcer characteristics*			
Localization of ulcer			
Toe	80 (51.3)	49 (62.7)	31 (39.8)
Metatarsal	23 (14.7)	8 (10.3)	15 (19.2)
Heal	12 (7.7)	7 (9.0)	5 (6.4)
Other	41 (26.3)	14 (18.0)	27 (34.6)
*Comorbidities*			
Cardiovascular disease^d^	42 (27.3)	19 (24.7)	23 (29.9)
Neuropathy^e^	102 (70.0)	51 (70.0)	51 (70.0)
Renal disease, GFR < 60	58 (37.2)	31 (39.7)	27 (34.6)
Retinopathy	53 (37.1)	23 (31.9)	30 (42.3)
*Ulcer endpoints*			
Healing	124 (79.5)	64 (82.1)	60 (76.9)
Amputation	15 (9.6)	4 (5.1)	11 (14.1)
Death	9 (5.8)	4 (5.1)	5 (6.4)
Not healed after 12 months	8 (5.1)	6 (7.8)	2 (2.6)

Data are shown as *n* (%) Percent of patients with valid values for categorical variables and mean ± SD for continuous variables

^a^In the current study we included all participants with at least one valid patient-reported outcome measure at baseline

^b^Education level: primary: up to 10 years of compulsory education, secondary: high school or vocational school and tertiary: college/university

^c^HbA1c measurements were reported using the International Federation of Clinical Chemistry units (mmol/mol) in addition to the derived NGSP units (%) upon attendance at the outpatient clinic

^d^Cardiovascular disease was defined as a history of angina pectoris, myocardial infarction or stroke

^e^Neuropathy: Neuropathy was defined as an abnormal pressure sensation evaluated with the 10 g monofilament and/or presence of symptoms and/or signs of peripheral nerve dysfunction

**Table 2:** Patient-reported outcomes comparing telemedicine versus standard outpatient care: the DiaFOTo study, Western Norway^a^

		Telemedicine (*n =* 78)					Standard Outpatient Care (SOC) (*n* = 78)				Intervention effect	
		Baseline		Follow-up	*p*-value		Baseline		Follow-up	*p*-value	Effect^b^	*p*-value
*Generic Quality of Life* measures^c^	n		n			n		n				
EQ-5D-5L, (0–1)	75	0.72 (0.3)	67	0.76 (0.2)	0.06	72	0.70 (0.3)	56	0.75 (0.2)	**0.02**	−0.01 (−0.08, 0.05)	0.66
EQ-VAS, (0–100)	74	59.0 (21.8)	68	60.4 (21.7)	0.53	73	57.7 (20.8)	57	63.5 (21.4)	**0.02**	−3.99 (−10.17, 2.19)	0.21
WHO-5, (0–100)	73	63.2 (21.0)	64	65.4 (19.8)	0.44	67	59.4 (21.5)	54	62.4 (20.6)	0.48	−0.67 (−6.74, 5.39)	0.83
HADS-A, (0–21)	77	4.3 (3.3)	66	4.5 (3.9)	0.89	78	4.6 (4.0)	58	4.7 (3.9)	0.43	−0.25 (−1.28, 0.78)	0.63
HADS-D, (0–21)	77	4.7 (3.3)	66	4.7 (3.9)	0.88	78	5.4 (4.1)	58	5.1 (3.5)	0.57	0.15 (−0.83, 1.1)	0.76
*Disease-specific Quality of Life* measures^c^												
PAID-20, mean (SD)	68	21.6 (18.6)	64	25.4 (20.0)	**0.04**	62	21.4 (18.3)	48	22.3 (18.0)	0.76	2.56 (−1.95, 7.06)	0.27
PAID-20 ≥ 40 (%)	68	14 (20.6)	64	11 (17.2)	0.31	62	8 (12.9)	48	7 (14.6)	0.85	−0.34 (−1.22, 0.54)	0.45
NeuroQOL weighted (1–15)												
Painful symptoms	75	4.0 (2.2)	65	4.1 (2.6)	0.97	73	4.2 (2.7)	58	4.2 (2.7)	0.89	0.06 (−0.77–0.88)	0.90
Reduced feeling	71	4.8 (3.7)	63	4.4 (3.3)	0.19	70	5.2 (3.3)	55	4.7 (3.3)	0.26	−0.03 (−1.06–0.99)	0.95
Diffuse sensorimotor symptoms	70	5.8 (4.2)	64	5.5 (4.3)	0.29	71	5.7 (4.0)	54	5.4 (3.7)	0.12	0.23 (−0.78–1.24)	0.66
ADL restrictions	69	7.2 (4.2)	58	6.5 (3.7)	0.19	**70**	8.2 (4.4)	52	6.3 (4.0)	**0.003**	1.05 (−0.65–2.75)	0.23
Interpersonal burden	70	5.5 (3.9)	59	5.1 (4.0)	0.18	71	5.3 (3.5)	50	4.7 (3.6)	0.20	0.02 (−1.18–1.22)	0.97
Emotional distress	75	4.0 (2.2)	63	4.1 (2.6)	0.97	73	4.2 (2.7)	53	4.2 (2.7)	0.89	0.06 (−0.77–0.88)	0.90

Data are shown as mean ± SD for continuous variables and *n* (%) percent for categorical variables

^a^In the current study we included all participants with at least one valid patient-reported outcome measure at baseline

^b^Regression coefficient for interaction between time and treatment group in linear mixed effects model for continuous outcome and generalized estimation equations (GEE) for binary outcomes

^c^Higher scores on EQ-VAS, or WHO-5 reflect better perceived health, health state or psychological well-being. Higher scores on EQ-5D, HADS-anxiety or –depression or PAID-20 reflect lower health-related quality of Life, more anxiety or depressive symptoms or more diabetes distress; Higher scores on the NeuroQOL reflect greater impact of foot ulcers on quality of life

### Outcome measures

The mean scores for generic measures (EQ-5D-5L, WHO-5, HADS) were relatively stable from baseline to post intervention in both treatment groups, with no significant intervention effect (Table 2). Within the standard treatment group, the mean EQ-5D-VAS score improved slightly (from 57.7 (SD 20.8) to 63.5 (SD 21.4), *P* = 0.02) but not in the TM group (from 59.0 (SD 21.8) to 60.4 (SD 21.7), *P* = 0.53).

Both disease-specific measures, PAID-20 and NeuroQOL, were also relatively stable and did not show an intervention effect (Table 1). Within the treatment groups, the mean PAID score increased from baseline to post intervention in the TM group (*P* = 0.04), indicating emotional problems. The proportion of patients with a PAID score of 40 or more decreased from 20.6% at baseline to 17.2% in the TM group (ns) and increased from 12.9 to 14.6% in the corresponding time period in the SOC group (ns). The mean weighted ADL restriction score (subscale from NeuroQOL) decreased within the standard treatment group, indicating less ADL restrictions (from 8.2 (4.4) at baseline to 6.3 (4.0) at follow-up, *P* = 0.003), but not in the TM group (from 7.2 (4.2) at baseline to 6.5 (3.7) at follow-up, *P* = 0.19) (Table 2). None of the additional analyses revealed significant differences between the treatment groups.

## Discussion

This is the first randomized controlled trial assessing the effect of a telemedicine intervention for DFUs, compared to SOC, designed primarily to study its effect on ulcer healing. Here we report the effect on self-reported health, well-being and QOL. PROMs were relatively stable from baseline to post-intervention in both treatment groups and the intervention did not affect the participants’ health, well-being or QOL.

As no previous studies have reported the effect of telemedicine follow-up on PROMs in patients with a DFU, we compare our findings to other relevant telemedicine studies. Telehealth as implemented in the Whole Systems Demonstrator Evaluation did not improve QOL or psychological outcomes for patients with chronic obstructive pulmonary disease (COPD), diabetes, or heart failure over 12 months [1]. In a sub-study, for those with COPD there was a consistent trend of improved health related QOL in the intention to treat analysis [21]. However, in the subsample of people with diabetes, the telemedicine intervention was not effective [22]. A systematic review including other studies of patients with COPD found mixed results on various QOL measures [23]. Another systematic review, on asynchronous and synchronous teleconsultation for diabetes care, concluded that neither synchronous nor asynchronous teleconsultations for diabetes care showed any significant differences between control (usual care) and intervention groups for QOL. Nevertheless, these studies found that increased intensity of contact between provider and patients were perceived as more supportive and generated more effective communication [24].

Possible mechanisms or pathways by which our telemedicine intervention might have improved PROMs include: (1) telemedicine might produce better DFU treatment outcomes (fewer adverse outcomes, quicker recovery time), which in turn result in better PROMs; and (2) patients might find telemedicine more supportive, independent of treatment outcome. In this study, there was a significantly lower proportion of amputation the first 12 months (*n* = 6 in TM, *n* = 13 in SOC, mean difference − 8.3, 95% CI –16.3, − 0.5%). DFU treatment outcomes (healing time and patient satisfaction) were similar for the TM and SOC groups. Therefore, we would not expect that different treatment outcomes would cause improved PROMs in the telemedicine group and the first proposed mechanism is thus unlikely to occur [9]. However, there was some evidence that telemedicine was perceived positively by participants. In general, patients experienced that if TM functions as intended, it can be an important additional tool [25]. Moreover, results from qualitative studies among healthcare personnel, conducted in conjunction with this RCT, indicated that using the TM intervention enhanced confidence among community nurses, as they perceived improvement in their wound care skills and facilitated more comprehensive DFU care [26–28]. The second proposed mechanism could thus have resulted in improved PROMs in the telemedicine group. Nevertheless, positive experiences among healthcare personnel and increased wound knowledge do not necessarily influence patients’ perception of health, well-being and QOL, which may explain why we did not find any effects on PROMs.

### Strengths and limitations

One strength of this study was the design; RCTs have been recommended to improve the evidence-base for treating DFUs in clinical practice and to facilitate changes in relevant care pathways [29]. Another strength was the real-life context as the study was performed in daily clinical practice at three sites. It has been suggested that there is a need for more research in TM interventions for well-defined patient groups in order to increase study validity [30]; in this study only participants with a DFU were included, not patients with other types of ulcers or other foot problems. Finally, this study used a broad range of PROMs, including disease-specific measures, to reduce the problems with insensitive or irrelevant outcome measures.

However, this study also has limitations. First, patients with a diagnosed mental disorder or cognitive impairment who might benefit most were excluded. More flexible health services may be especially beneficial for patients with more complex illness living in nursing homes or having mental problems and those having difficulties traveling to a hospital. However, most of these frail elderly patients were excluded from participation in the current trial as they were unable to give informed consent. Further, the sample size estimation was done for the primary endpoint (ulcer healing time) and we cannot rule out the possibility that we failed to detect small positive or negative intervention effects because of Type II error. Although it would be of interest to test if the effect of the intervention on PROs differed between patients with healed DFU and patients with remaining DFU and/or amputations, the sample size of the current study was too small to be able to detect such possible effect modifications. Participants in the study had a relative longstanding diabetes and most likely established self-management routines. It is possible that it was too optimistic to expect that a relative short telemedicine intervention should affect patient reported outcomes. As to user involvement, one patient was part of the research team, however we could have invited a larger group of patients as co-workers leading to a more systematic co-production and an intervention perhaps better tailored to patients’ needs [24, 31].

## Conclusions

Telemedicine technology has emerged as a relevant alternative to usual care for people with DFUs, facilitating flexible healthcare services and close cooperation between levels of healthcare services [9]. The intervention did not have a significant effect on DFU treatment outcomes or PROMs; measures of health, well-being and QOL were relatively stable from baseline to post-intervention in both treatment groups. Although the intervention did not yield short-term benefit for the participants, it is notable that it was also was not associated with worse outcomes. TM is as effective as face-to-face care, while having greater reach. Future studies may reveal that it is possible to modify the telemedicine intervention to more accurately fit settings and contexts in order to generate both improved ulcer healing and patient-reported outcomes.

## Supplementary information

**Additional file 1: Fig. S1** Supplementary appendix: The Consort flow chart for the primary study

**Additional file 2: Table S1** Supplementary appendix: Completion rates at baseline and follow-up in the current study: the DiaFOTo study, Western Norway^1^. ^1^ The study population in the current study is defined as patients with at least one valid patient-reported outcome measure (PROM) at baseline (*n* = 78 in the telemedicine group and *n =* 78 in the standard outpatient care group, *n* = 156 in total). *n* = 182 among the participants in the main study. **Table S2** Supplementary appendix: Unweighted NeuroQOL scores comparing telemedicine versus standard outpatient care: the DiaFOTo study, Western Norway. *Higher scores on the NeuroQOL reflect greater negative impact of foot ulcers on quality of life

## Data Availability

The datasets generated and/or analysed during the current study are not publicly available due to personal data protection legislation but are available from the corresponding author on reasonable request.

## Abbreviations

TM: Telemedicine
SOC: Standard outpatient care
DFU: Diabetes Foot Ulcer
QOl: Quality of Life
HRQOL: Health related Quality of Life.
EQ-5D-5L: Euro-QOL five-dimension questionnaire
HADS: The Hospital Anxiety and Depression Scale (HADS)
PAID: Problem Areas in Diabete
NeuroQOL: Neuropathy- and Foot Ulcer–Specific Quality of Life Instrument
PROMs: Patient-reported outcome measures
GEE: Generalized estimation equations
