# Supplementary figures and images for: Effect of a telemedicine intervention for diabetes-related foot ulcers on health, well-being and quality of life: secondary outcomes from a cluster randomized controlled trial (DiaFOTo)
Source: BMC Endocr Disord. 2020 Oct 21;20:157. doi: 10.1186/s12902-020-00637-x (PMC7580005; doi:10.1186/s12902-020-00637-x)

**Figure S1** Consort flow diagram for the primary study [9]


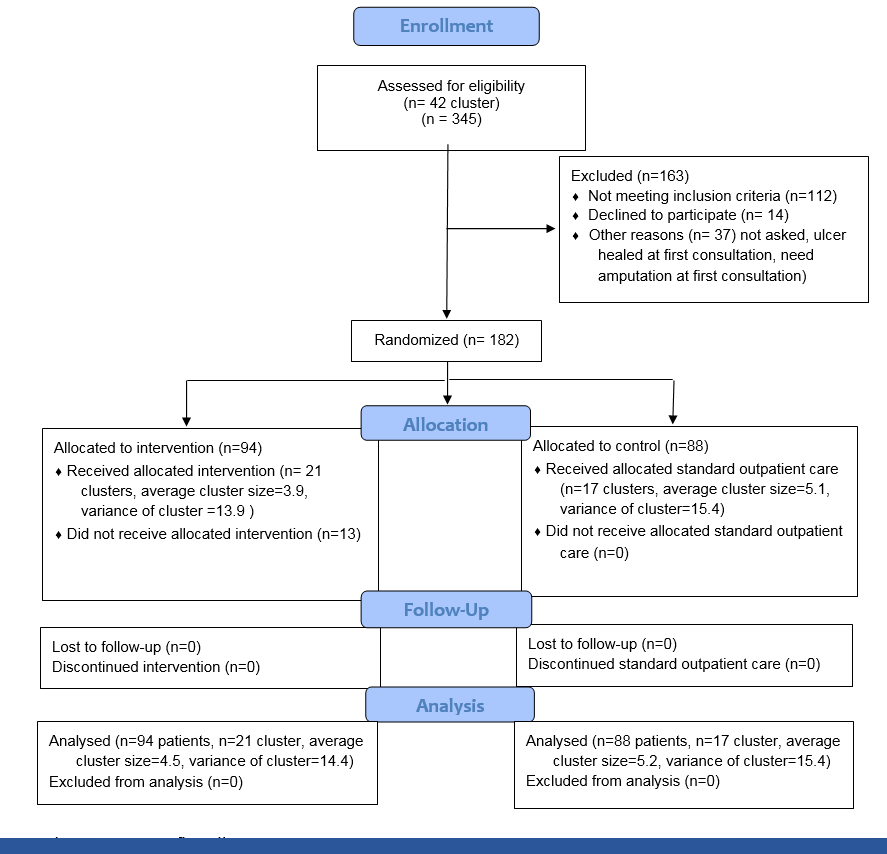

Supplement: Supplementary file 1 — Additional file 1: Fig. S1 Supplementary appendix: The Consort flow chart for the primary study [file 12902_2020_637_MOESM1_ESM.docx]
