# Supplementary material for: Effect of a telemedicine intervention for diabetes-related foot ulcers on health, well-being and quality of life: secondary outcomes from a cluster randomized controlled trial (DiaFOTo)
Source: BMC Endocr Disord. 2020 Oct 21;20:157. doi: 10.1186/s12902-020-00637-x (PMC7580005; doi:10.1186/s12902-020-00637-x)
Supplement: Supplementary file 2 — Additional file 2: Table S1 Supplementary appendix: Completion rates at baseline and follow-up in the current study: the DiaFOTo study, Western Norway1. 1 The study population in the current study is defined as patients with at least one valid patient-reported outcome measure (PROM) at baseline (n = 78 in the telemedicine group and n = 78 in the standard outpatient care group, n = 156 in total). n = 182 among the participants in the main study. Table S2 Supplementary appendix: Unweighted NeuroQOL scores comparing telemedicine versus standard outpatient care: the DiaFOTo study, Western Norway. *Higher scores on the NeuroQOL reflect greater negative impact of foot ulcers on quality of life [file 12902_2020_637_MOESM2_ESM.docx]

**Table S1** Supplementary appendix: Completion rates at baseline and follow-up in the current study: the DiaFOTo study, Western Norway^1^

|  |  | Baseline | |  | Follow-up | |
| --- | --- | --- | --- | --- | --- | --- |
|  |  | N with | Percent |  | N with | Percent |
|  |  | valid scores | completion |  | valid scores | completion |
| EQ-5D-5L | | 147 | 80.8 |  | 123 | 67.6 |
| EQ-VAS | | 147 | 80.8 |  | 125 | 68.7 |
| WHO-5 | | 140 | 76.9 |  | 118 | 64.8 |
| HADS-A, | | 155 | 85.2 |  | 124 | 68.1 |
| HADS-D | | 155 | 85.2 |  | 124 | 68.1 |
| PAID-20 | | 130 | 71.4 |  | 112 | 61.5 |
| NeuroQOl symptoms | |  |  |  |  |  |
|  | Painful symptoms | 150 | 82.4 |  | 123 | 68.1 |
|  | Reduced feeling | 141 | 77.5 |  | 124 | 68.1 |
|  | Diffuse sensorimotor symptoms | 149 | 81.9 |  | 124 | 68.1 |
|  | ADL restrictions | 146 | 80.2 |  | 114 | 62.6 |
|  | Interpersonal burden | 149 | 81.9 |  | 116 | 63.7 |
|  | Emotional distress | 151 | 83.0 |  | 121 | 66.5 |
| At least one valid PROM | | 158 | 86.8 |  | 127 | 69.8 |

^1^ The study population in the current study is defined as patients with at least one valid patient-reported outcome measure (PROM) at baseline (n=78 in the telemedicine group and n=78 in the standard outpatient care group, n=156 in total). n=182 among the participants in the main study.

**Table S2** Supplementary appendix: Unweighted NeuroQOL scores comparing telemedicine versus standard outpatient care: the DiaFOTo study, Western Norway

|  | | Telemedicine  (n=69) | | |  | Standard Outpatient Care (SOC)  (n=58) | | |  | Intervention effect  (Difference in difference analyses) | |
| --- | --- | --- | --- | --- | --- | --- | --- | --- | --- | --- | --- |
|  | | Baseline | Follow-up | p-value |  | Baseline | Follow-up | p-value |  | Effect* | p-value |
| NeuroQOL, unweighted | |  |  |  |  |  |  |  |  |  |  |
|  | Painful symptoms | 2.06 (0.76) | 2.07 (0.83) | 0.93 |  | 2.15 (0.91) | 2.13 (0.85) | 0.87 |  | 0.06 (-0.77-0.87) | 0.90 |
|  | Loss of sensation | 1.62 (0.59) | 2.40 (1.25) | **<0.001** |  | 1.68 (0.59) | 2.42 (1.26) | **<0.001** |  | 0.008 (-0.37-0.38) | 0.99 |
|  | Diffuse sensory motor symptoms | 3.41 (1.32) | 2.51 (1.33) | **<0.001** |  | 3.45 (1.25) | 2.43 (1.11) | **<0.001** |  | 0.12 (-0.48-0.71) | 0.70 |
|  | ADL restrictions | 3.07 (1.37) | 2.78 (1.24) | 0.13 |  | 3.35 (1.24) | 2.64 (1.23) | **<0.001** |  | 0.33 (-0.22-0.88) | 0.24 |
|  | Interpersonal burden | 2.45 (1.21) | 2.28 (1.22) | 0.05 |  | 2.37 (1.17) | 2.11 (1.09) | 0.07 |  | -0.008 (-0.34-0.32) | 0.96 |
|  | Emotional distress | 2.32 (1.21) | 2.28 (1.17) | 0.51 |  | 2.31 (1.20) | 2.26 (1.16) | 0.50 |  | 0.006 (-0.28-0.29) | 0.95 |

*Higher scores on the NeuroQOL reflect greater negative impact of foot ulcers on quality of life

s
